# Novel Antifungal Activity for the Lectin Scytovirin: Inhibition of *Cryptococcus neoformans* and *Cryptococcus gattii*

**DOI:** 10.3389/fmicb.2017.00755

**Published:** 2017-05-09

**Authors:** Tyler H. Jones, Erin E. McClelland, Hana McFeeters, Robert L. McFeeters

**Affiliations:** ^1^Department of Chemistry, University of Alabama in Huntsville, HuntsvilleAL, USA; ^2^Department of Biology, Middle Tennessee State University, MurfreesboroTN, USA

**Keywords:** Scytovirin, *Cryptococcus neoformans*, *Cryptococcus gattii*, novel antifungal, lectin, cryptococcosis

## Abstract

Pathogenic cryptococci are encapsulated yeast that can cause severe meningoencephalitis. Existing therapeutic options are dated and there is a growing need for new alternative antifungal agents for these fungi. Here we report novel inhibition of pathogenic cryptococci by the antimicrobial lectin Scytovirin. Inhibition was most potent against *Cryptococcus neoformans* var *neoformans* and *C. gattii*, with MFC values of 500 nM. Scytovirin binding was localized to the cell wall and shown to affect capsule size and release. No effect was observed on melanization or with cells grown in the presence the cell wall stressor Congo red. Synergy with existing antifungals was indicated, most strongly for amphotericin B. Overall, Scytovirin serves as a much needed new avenue for anticryptococcal development.

## Introduction

Pathogenic cryptococci are encapsulated basidiomycotic yeast that cause life-threatening infections worldwide. An estimated 1 million cases of cryptococcal meningitis occur among those with HIV/AIDS each year, resulting in nearly 625,000 deaths (Park et al., 2009). Based on genetic characteristics and serologic properties of capsular polysaccharides, multiple varieties and serotypes have been defined. *Cryptococcus neoformans* var *grubii* (serotype A) and var *neoformans* (serotype D) (Franzot et al., 1999; Lengeler et al., 2001) mainly infect immunocompromised hosts. *C. gattii*, formerly *C. neoformans* var. *gattii* (serotypes B and C), infect immunocompromised as well as immunocompetent hosts. Certain epidemiological properties have been associated with individual serotypes. Generally, serotypes A, D, and hybrid AD are found worldwide while *C. gattii* is mainly found in tropical and subtropical regions (Casadevall and Perfect, 1998; Kwon-Chung et al., 2000), with more recent emergence in the U.S. Pacific Northwest (Bartlett et al., 2008, 2012; Datta et al., 2009). Serotype D infections are more prevalent in Western Europe (Mitchell and Perfect, 1995; Dromer et al., 1996a; Bennett et al., 1977), accounting for 21% of cryptococcosis cases in France (Dromer et al., 1994, 1996b) and about 30% is Europe (Cogliati et al., 2016), although it has been indicated that many of these strains are AD hybrids (van Elden et al., 2000; Gago et al., 2017). Serotype D infections are also more likely to occur in older patients than serotype A infections (Dromer et al., 1994, 1996b). Thus, understanding differences in serotypes and how they relate to pathogenesis is crucial.

The current gold standard treatment for cryptococcal meningitis is a combination of broad spectrum antifungals amphotericin B (AMB) and flucytosine (5FC), with long term maintenance doses of fluconazole (FLC) (Perfect et al., 2010). Despite being effective, this regimen can lead to dangerous side effects and the inevitable development of resistant strains. Therefore, new and improved cryptococcal treatments are of critical clinical need.

The fungal cell wall has been long recognized as a promising target for antifungal development (Georgopapadakou and Tkacz, 1995; Selitrennikoff and Nakata, 2003). For pathogenic cryptococci, the cell wall is composed of a chitin rich layer close to the cell membrane surrounded by a polysaccharide network composed of β-1,3- and β-1,6-glucans, xylomannans, and galactomannans. Similar to other yeast organisms, the cell wall of *Cryptococcus* also contains α-1,3-glucans that anchor the cryptococcal capsule (Reese and Doering, 2003). The cryptococcal cell wall also contains a number of N- and O-linked glycoproteins cross-linked to the carbohydrate matrix which have a variety of roles and serve as structural elements (Bowman and Free, 2006; Doering, 2009). Typical for fungal glycosylation, the N-linked glycoproteins contain high mannose glycans (Deshpande et al., 2008). Proper glycosylation and the presence of properly glycosylated proteins are absolutely vital for fungal well-being with defects in N-glycosylation having been shown to induce apoptosis (Hauptmann et al., 2006). Thus, targeting glycans within the cell wall is an attractive option for new antifungal development.

The cyanobacterial lectin Scytovirin contains two carbohydrate binding domains (McFeeters et al., 2007a) and is highly specific for Man α (1-2)Man α (1-6)Man α (1-6)Man tetramannose (Adams et al., 2004; McFeeters et al., 2007b), an ever-present building block of viral and fungal high mannose glycans (Deshpande et al., 2008; Martinez-Duncker et al., 2014). Scytovirin is known for antiviral activity against HIV-1, Ebola, and Hepatitis C (Bokesch et al., 2003; McFeeters et al., 2012; Takebe et al., 2013; Garrison et al., 2014). Favoring therapeutic development, Scytovirin demonstrates very low toxicity in both human hepatocyte carcinoma cell lines and mice models. Preliminary studies using Huh7.5.1 cells showed low toxicity and a high selectivity index (Takebe et al., 2013). Further, no toxic effects were seen in murine models where mice were given up to 30 mg/kg/day of Scytovirin (Garrison et al., 2014). Also, no toxicity was reported up to 200x the EC_50_ found in anti-Ebola studies (Garrison et al., 2014). While an active antimicrobial *in vivo*, the half-life of Scytovirin may indicate that it is being rapidly metabolized, an area of interest for future development.

Herein we report novel antifungal activity for Scytovirin against pathogenic cryptococci. The most potent inhibition was observed against serotype D and *C. gattii* strains with MFC values of 500 nM compared to serotype A strains which demonstrated MFCs of approximately 20 μM. Fluorescence confocal microscopy localized Scytovirin to the cryptococcal cell wall where it was shown to affect capsule release. No effect on melanization or growth in the presence of the cell wall stressor Congo red was observed. Scytovirin inhibition was synergistic with existing antifungals, most potently for AMB. While potentially explaining the presence of Scytovirin in the cyanobacterium *Scytonema varium*, these findings point to new utility for Scytovirin as an anticryptococcal agent. Already under development for antiviral applications, Scytovirin represents a promising new avenue for development of a much needed anticryptococcal agent and a potential tool for discerning greater insight into *Cryptococcus* serotype differences.

## Materials and Methods

### Scytovirin Expression and Purification

Scytovirin was produced and purified as described previously (McFeeters et al., 2007b, 2013) using recombinant expression in Origami^TM^ *E. coli* cells and subsequent metal chelation chromatography and HPLC purification. Purified Scytovirin was lyophilized and resuspended in phosphate buffered saline (PBS).

### Strains

Cryptococcal strains used were: (serotype A) H99S (Janbon et al., 2014), KN99a, KN99α, and Botswana clinical isolates B5, B15, B18, and B45 (Bisson et al., 2008); (serotype D) 24067, JEC21, B3501, B3502, Cap67, and clinical isolates AD3-80, AD5-33, AD7-17, AD11-23, AD11-75, AD11-77, AD12-15, AD12-22, and AD12-27 (Dromer et al., 2007; Desnos-Ollivier et al., 2015). Two clinical strains of *C. gattii* were also tested: R265 and R272 (Ngamskulrungroj et al., 2011). All strains were grown from frozen stock in Yeast Peptone Dextrose (YPD) broth to mid-log phase (36–42 h) at 37°C and then washed 3x with PBS.

### Minimum Fungicidal Concentration

The minimum fungicidal concentration was determined using a modified version of the CLSI M27-A2 protocol (CLSI, 2002). For each strain, a total of 2 × 10^3^ cells were resuspended in 900 μl of RPMI + 0.1 M MOPS (pH = 7.0) and aliquoted into sterile 5 mL culture tubes. The initial inoculum for each strain was plated on YPD to determine CFU. Different concentrations of Scytovirin in PBS were added in 100 μl to reach final concentrations of 0, 0.5, 1.0, 1.3, 1.5, 2.6, 5, 10, and 20 μM. The tubes were incubated at 37°C for 72 h, shaking at 150 rpm. After 72 h, serial dilutions of each tube were plated on YPD. In addition, 100 μl of an appropriate dilution was plated on YPD to determine CFU. We reasoned that *C. neoformans* would be maintained in a more aerobic environment by shaking (not settling to the bottom of a plate well) and provide a better indicator of inhibition. Also, measuring CFU provided a better indication of live cells. The concentration of Scytovirin that resulted in greater than 95% inhibition was considered the MFC (Supplementary Figure 1). It was confirmed that Scytovirin inhibited *C. neoformans* in a 96-well plate format and absorbance readings correlated with broth macrodilution, although typically resulted in lower MIC values. That is a 1 μM MFC in the reported fungal kill assay correlated to an approximate 300 μM MIC measured from 96-well plates.

### Capsule Size

Capsule size *in vitro* was measured as described (Zaragoza et al., 2003). Briefly, 2 × 10^5^ cells/mL of strains H99S (serotype A), 24067 and B3502 (serotype D) were added to DMEM in the presence of 0, 1, and 10 μM Scytovirin and incubated at 37°C + 5% CO_2_ for 18 h. Cells were collected, suspended in 5 μl PBS and added to a microscope slide with India Ink. Cells were imaged on a Zeiss Axio Scope A1 inverted microscope with a 100X objective. For each strain, pictures of 50 cells were taken. The diameter of the cell body and capsule were measured using Zeiss Axiovision software. Capsule radial length was calculated by subtracting the cell body diameter from the diameter of the entire cell plus capsule and dividing by 2. Cells were also imaged before capsule induction as a control to ensure that capsule production was induced. The experiment was repeated three times.

### Glucuronoxylomannan Release

To determine if the strains differed in ability to release capsular glucuronoxylomanan (GXM) into the medium in the presence of Scytovirin, capsule production was induced in DMEM as before. The next day, the supernatant was collected and the concentration of GXM in the media was measured by capture ELISA as previously described (Casadevall et al., 1992). Experiments were repeated in triplicate. The following antibodies were used: goat anti-mouse unlabeled IgM, anti-GXM Ab 2D10, anti-GXM ab 18B7, and labeled goat anti-mouse IgG1-AP.

### Melanin Production and Cell Wall Stress

To determine if Scytovirin affected melanin production or growth in the presence of cell wall stressors, Scytovirin (0, 1, 10, and 20 μM) was added to L-Dopa plates and YPD plates containing 0.5% Congo red. Log-phase cultures of serotype A strain H99S, and serotype D strains B3501, B3502, Cap67, AD-3-80, and AD11-23 were washed 3X with PBS, diluted to 1 × 10^6^ cells/mL and serial dilutions were spotted onto L-Dopa and 0.5% Congo red plates. Plates were incubated at 37°C for 2–7 days and the growth of different spot dilutions quantified.

### Antibodies and Immunofluorescence Microscopy

Serotype D strains 24067, B3502, and Cap67, and serotype A strain H99S cells were labeled with the cell wall stain uvitex2B (Polysciences Inc., 1 μg/mL), 200 μg/mL AlexaFluor 568-conjugated Scytovirin, and 10 μg/mL AlexaFluor 488-conjugated IgM capsular antibody 12A1. Emissions from 410 to 480 nm (uvitex 2B), 495–525 nm (AlexaFluor 488), and 578–603 nm (AlexaFluor 568) were visualized using a Zeiss laser scanning confocal microscope. Images were processed with ImageJ (Abramoff et al., 2004).

### Chitin Binding Assay

The assay was performed in triplicate as previously described (Vaaje-Kolstad et al., 2005). Briefly, aliquots of Scytovirin (5 mg/mL) and chitin (MP Biochemicals, 20 mg/mL) were combined in PBS, pH 7.4, to produce 0.5 mL reaction mixtures containing 100 μg Scytovirin and 1 mg chitin. Reaction mixtures as well as control tubes containing only 100 μg Scytovirin or 1 mg chitin were incubated with continuous vortexing at 25°C for a given period of time. At defined time points between 5 min and 20 h, chitin was pelleted by centrifugation and the concentration of Scytovirin in the supernatant was determined by UV absorbance at 280 nm. The spectrophotometer was blanked using supernatant from a chitin only control tube. Additionally, supernatant samples were taken at each time point and analyzed by SDS-PAGE.

### *In Vitro* Interactions with Common Antifungals

*Cryptococcus neoformans* strain 24067 was used to determine potential synergistic interactions of Scytovirin with AMB, 5FC, and FLC. Interactions were evaluated using broth microdilution checkerboard assays and FIC indexing as well as response surface modeling (Greco et al., 1995). The FIC index was defined as follows: (MFC of drug A, tested in combination)/(MFC of drug A, tested alone) + (MFC of drug B, tested in combination)/(MFC of drug B, tested alone). Since FIC indices calculated at the 95% MFC cutoff varied significantly, a 50% inhibition level, or MFC-2 (Te Dorsthorst et al., 2002), was used. For surface response modeling, the entire dataset was modeled using the response surface approach (Greco et al., 1995; Te Dorsthorst et al., 2002) and the interaction coefficient ICα determined with 95% confidence bounds. Data were processed using MATLAB R2016a (The MathWorks, Inc.). An 8 mg/mL stock solution of AMB (Alfa Aesar) was prepared in DMSO. Stock solutions of 20 mg/mL 5FC (Alfa Aesar) and FLC (Acros Organics) were prepared in distilled deionized H_2_O. Subsequent dilutions were made in RPMI/MOPS pH 7.0. In each experiment, final Scytovirin concentrations ranged from 100 μg/mL to 7.6 × 10^-4^ μg/mL (10.3 μM to 78 pM). Final concentrations of AMB tested ranged from 8 μg/mL to 6.1 × 10^-5^ μg/mL, while concentrations of both 5FC and FLC ranged from 10 μg/mL to 7.6 × 10^-5^ μg/mL. *C. neoformans* 24067 cells were grown from frozen stock in YPD broth for 36–42 h at 37°C and then washed 3x and re-suspended in PBS before dilution into RPMI/MOPS. Tests were performed in black walled 96-well microplates with clear, flat bottoms. In each well, 50 μL of tested antifungal and 50 μL Scytovirin were combined at four times the final concentration in abscissa and ordinate dimensions, respectively. Hundred microliters of 4,000 cells/mL inoculum in RPMI/MOPS was added to each well. Plates were incubated at 37°C for 40 h before 20 μL of PrestoBlue (ThermoFisher) was added to each well and allowed to incubate for 8 h. Fluorescence excitation was at 560 nm and emission was read at 590 nm using a SpectraMax M2 microplate reader and SoftMax Pro software (VWR). Drug-free control plates were used as positive growth controls. Plates with RPMI/MOPS only or *C. neoformans* inoculum with excess AMB, which displayed no detectable fluorescence difference, were used as negative growth controls.

### Statistics

Capsule diameter was analyzed using the nonparametric Wilcoxon Rank Sums test while a multivariate analysis of variance with simple effects was used to test for GXM release. For all tests, *p*-values <0.05 were considered significant.

## Results

### Scytovirin Inhibits Cryptococcal Growth

Scytovirin binds high mannose moieties and has high specificity for Man4 (Adams et al., 2004; McFeeters et al., 2007b, 2013). Cryptococci produce highly mannosylated proteins and utilize mannose as a building block for their distinctive capsules. Therefore, Scytovirin was tested for inhibitory activity. Initially, cryptococcal susceptibility to Scytovirin was tested on the *C. neoformans* laboratory strains H99S (serotype A) and 24067 (serotype D). Scytovirin more potently inhibited 24067 cells (MFC of 1 μM) and more weakly inhibited H99S cells (MFC between 50 and 20 μM). To better gauge the ability of Scytovirin to inhibit pathogenic cryptococci, 23 strains covering 3 serotypes were tested, see **Table 1**. Having MFC values of 500 nM, the most potently inhibited serotype D strains were JEC21, B3502, and the clinical isolate AD12-27. The MFC of most other serotype D strains ranged from 1 to 5 μM, with one ranging to 20 μM. Representative *C. gattii* strains R265 and R272 were inhibited by Scytovirin, but the likely parent strain (R272) was more susceptible with an MFC of 500 nM. In general, serotype A strains were less susceptible. Botswana clinical isolates B5, B15, and B45 were most susceptible with MFC values of 20 μM. While their MFCs were greater than 20 μM, strains B18, H99S, and KN99a showed greater than 90% growth inhibition and KN99α greater than 75% growth inhibition in the presence of 20 μM Scytovirin.

**Table 1:** Scytovirin MFC for pathogenic cryptococci.

Species/serotype	Strain	MFC (μM)
*Cryptococcus neoformans* serotype D	24067	1
	JEC21	0.5
	B3501	20
	B3502	0.5
	Cap67	5
	AD3-80	5
	AD5-33	5
	AD7-17	5
	AD11-23	10
	AD11-75	10
	AD11-77	5
	AD12-15	10
	AD12-22	5
	AD12-27	0.5

*C. neoformans* serotype A	H99S	>20
	KN99A	>20
	KN99α	>20
	B5	20
	B15	20
	B18	>20
	B45	20

*C. gattii*	R265	10
	R272	0.5


### Scytovirin Localizes to the Cell Wall

**FIGURE 1:**
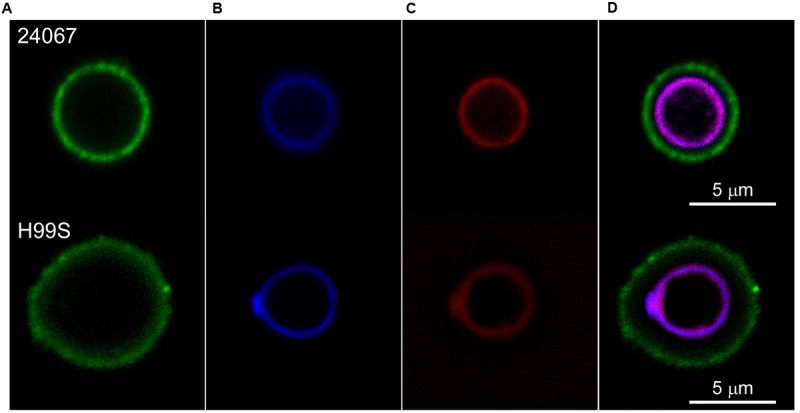
**Scytovirin Localization to the *C. neoformans* Cell Wall.** *Cryptococcus neoformans* serotype D 24067 (top) and serotype A H99S cells (bottom) were labeled with AlexaFluor488 conjugated 12A1 antibody against the capsule **(A)**, Uvitex B blue cell wall stain **(B)**, and AlexaFluor568 conjugated Scytovirin **(C)**. The overlay **(D)** shows that Scytovirin localizes to the cell wall of *C. neoformans*.

*Cryptococcus neoformans* were exposed to fluorescently labeled Scytovirin and localization was determined by fluorescence confocal microscopy. Fluorescently labeled capsular antibodies and cell wall stain aided in localization. From visualization at variable time intervals, it was observed that Scytovirin quickly assimilated on the cell wall. No fluorescence overlap of Scytovirin was detected with any of the capsular antibodies tested (**Figure 1**). To determine whether susceptibility was correlated with Scytovirin binding to the cell wall, additional experiments were performed with strains of varying MFC values. It was found that in all cases (B3501, B3502, cap67, and H99S) Scytovirin binding was localized to the cell wall, irrespective of serotype.

### Scytovirin Affects Capsule Size and Prevents Release of Capsular Polysaccharides

**FIGURE 2:**
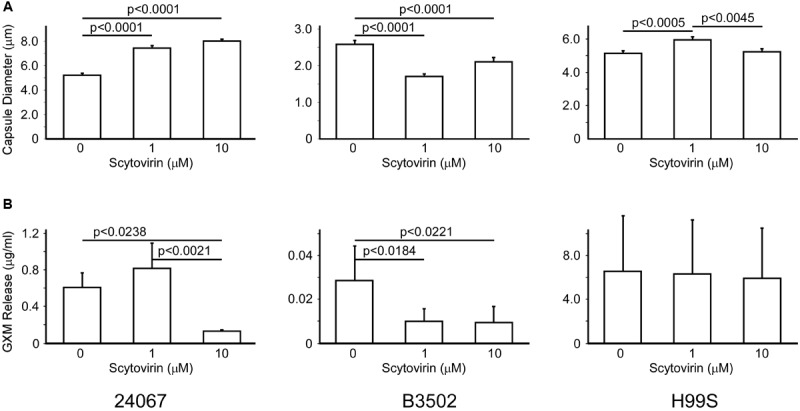
**Capsule Production and Release upon Treatment with Scytovirin.** Changes in capsule size **(A)** and GXM release **(B)** of serotype D strains 24067, B3502, and serotype A strain H99S treated with increasing concentrations of Scytovirin. Error bars depict standard error of the mean and data are representative of three independent experiments. Supplementary Figure 2 shows the one-way analysis.

To determine if the inhibitory activity of Scytovirin affected production of the capsule, strains 24067, B3502 (serotype D), and H99S (serotype A) were induced for capsule production in the absence or presence of two concentrations of Scytovirin. Interestingly, increasing concentrations of Scytovirin were associated with significant increases in capsule diameter for strain 24067, significant decreases in capsule diameter for strain B3502, and a small, but significant, increase in diameter for strain H99S (only at 1 μM) (**Figure 2A**). The largest changes were a 50% increase at 10 μM Scytovirin for strain 24067, a 35% decrease at 1 μM Scytovirin for strain B3502, and a 15% increase at 1 μM Scytovirin for strain H99S. The same trend is found when Scytovirin concentrations closest to each strains respective MFC were compared (1 μM is equal to the MFC for 24067, 1 μM or twice the MFC for B3502, and 10 μM or approximately one half the MFC for H99S).

To determine if Scytovirin affected release of capsular polysaccharides, strains 24067, B3502, and H99S were induced for capsule production in the presence or absence of Scytovirin as above. After overnight culture growth, the supernatant was collected and used to measure amounts of released polysaccharides. For both serotype D strains, Scytovirin concentrations 10-fold or greater than the MFC significantly inhibited release of capsular polysaccharides, which was attributed to fungicidal activity. Near the MFC, capsule release showed a potentially modest increase for 24067 but a significant decrease for B3502. No change in capsule release was observed for H99S (**Figure 2B**). Strain H99S was less susceptible to Scytovirin inhibition and in the absence of Scytovirin released 5- and 100-fold more capsule than strains 24067 and B3502, respectively, potentially mitigating any effects.

### Scytovirin Does Not Affect Cell Wall Integrity or Melanin Production

Scytovirin did not have any effect on cell wall integrity in the presence of 0.5% Congo red with control plates showing the same degree of growth as those with cell wall disrupting dye present for all strains tested (data not shown). Since Congo red binds β-glucans and interferes with the construction of the cell wall (Ram and Klis, 2006), this data suggests that Scytovirin does not bind β-glucans, as expected. Additionally, Scytovirin did not have any effect on melanization after 7 days incubation at 37°C with the same degree of melanization (darkening of conlonies) observed with and without Scytovirin present for all strains tested.

### Scytovirin Does Not Interact with Chitin

**FIGURE 3:**
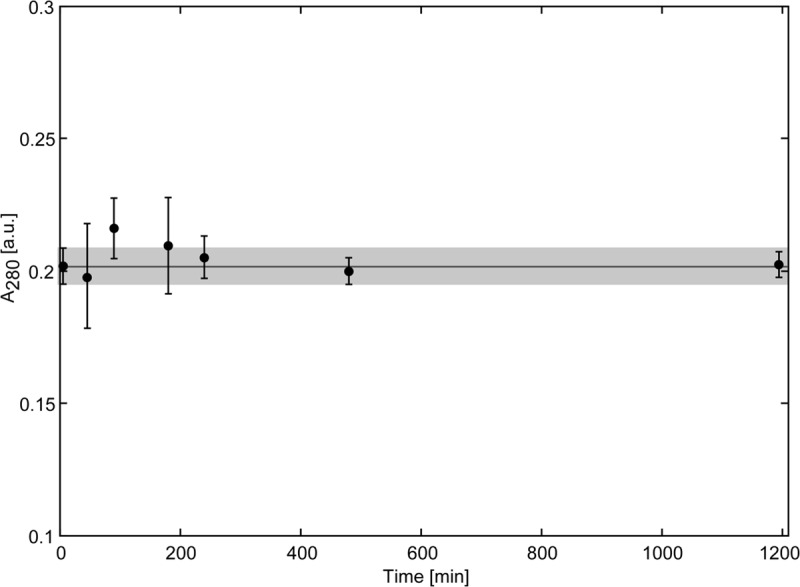
**Scytovirin Does Not Bind Chitin.** The concentration of Scytovirin in the supernatant of a reaction mixture containing chitin and Scytovirin remains constant even upon prolonged incubation. The values of Scytovirin concentration in units of absorbance are averages of three independent measurements. Error bars show the average deviation. The horizontal line indicates the average Scytovirin concentration in the control experiments without chitin and the gray box marks the range of ±1 SD from the average Scytovirin concentration in the control experiment.

Biochemical characterization and structural studies have indicated similarity of Scytovirin to chitin binding proteins. With the strong accumulation of Scytovirin in the cryptococcal cell wall, the ability of Scytovirin to bind chitin was determined. Scytovirin did not show any interaction with chitin, **Figure 3**. Even over the course of 20 h, no binding or adsorption to chitin was observed, in agreement with previous chitin microcolumn data (Bokesch et al., 2003).

### Scytovirin Exhibits Synergy with Existing Antifungals

Using 24067 cells, the combinatorial effects of Scytovirin with the commonly used antifungals AMB, 5FC, and FLC were investigated. When the MFC at 95% inhibition (MFC-0) was used for the calculation, results varied significantly. This was in part due to the steep response of fungal inhibition of AMB, declining from no inhibition to complete inhibition within a factor of 4 in terms of AMB concentration, or with just three wells on the plate. Therefore, FIC indices were calculated at 50% inhibition (MFC-2), yielding more consistent results (Te Dorsthorst et al., 2002). The FIC index using MFC-2 was 0.3 for the combination of AMB and Scytovirin (Supplementary Figure 3). Similarly, FIC indexing values for 5FC and FLC in combination with Scytovirin were 0.56 and 0.75, respectively. To better evaluate synergy, ICα values from response surface modeling were also determined using a standard checkerboard arrangement of orthogonal increasing inhibitor concentrations. Data from the entire 96-well plate were used to create a 3-dimensional response surface map With ICα determined from fitting the response surface map to the surface equation relating inhibitor concentrations and the effect at those concentrations (Te Dorsthorst et al., 2002). Positive ICα values indicate synergy, negative values indicate antagonism, and values close to zero indicate no interaction. AMB presented the strongest synergistic interaction with Scytovirin, having an ICα of 2.27. 5FC and FLC both indicated lesser degrees of synergy than AMB, having ICα values of 0.72 and 0.60, respectively.

## Discussion

Throughout their existence, organisms have developed multiple means to protect themselves from various environmental predators. The specific nature of the inhibitory molecules varies, but peptides and proteins have become heavily involved with utilization spanning the spectrum from bacteria, through fungi, to higher eukaryotes (McFeeters and McFeeters, 2012; Nakatsuji and Gallo, 2012; Rathi et al., 2012). Plants were the first organisms found to use carbohydrate binding proteins, or lectins, as part of their defense mechanisms (Sharon and Lis, 2004). Since then, many lectins have been identified from a number of organisms, including cyanobacteria.

A subset of lectins from cyanobacteria has been shown to have antiviral properties linked to their ability to recognize and bind N-linked high mannose glycans on viral envelope glycoproteins (Huskens and Schols, 2012). Though lectins are generally not well-known for antifungal activity, here we report the high mannose binding cyanobacterial lectin Scytovirin has the ability to inhibit pathogenic cryptococci. Our results indicate that Scytovirin, and potentially other cyanobacterial lectins, could be employed as important defense mechanisms to recognize and inhibit fungal pathogens. This finding potentially explains the presence of these lectins in cyanobacteria and other ancient organisms.

From the localization studies, it seems that the target(s) recognized by Scytovirin is not found in the largely GXM outer capsule of *C. neoformans*, but near the cell wall. The recognition of high mannose glycans on N-linked glycoproteins is further supported by of the inability of Scytovirin to bind chitin. In addition to simple recognition, the interaction of Scytovirin with *C. neoformans* seems to interfere with normal functioning. In this case, lectin binding affects capsule production and release of capsular polysaccharides, the pathogens most important virulence factor. Beyond localizing to the cell wall and affecting capsule production and release, little is known about how Scytovirin inhibits pathogenic cryptococci. The *C. neoformans* capsule is synthesized in the cytoplasm, packaged into vesicles for release at the cell wall (Rodrigues et al., 2007), and is thought to be extracellularly transported via secretory pathways (Yoneda and Doering, 2006; Panepinto et al., 2009). The different effects of Scytovirin on capsular polysaccharides from various strains suggest multiple possibilities. For example, Scytovirin binding may impact vesicle release or action of the associated secretory pathway explaining the correlation between changes in capsule release for Serotype D strains at or below the MFC (i.e., at 1 μM). Above the MFC, the fungicidal activity of Scytovirin kills the cells and thus likely halts capsule release. This also provides an explanation for inhibition of H99S, which releases considerably more capsule than the other strains tested. H99S displays minimal change in capsule release in the presence of Scytovirin presumably due to the elevated level of production and secretion. Further, the increases in capsule diameter observed for strains 24067 and H99S may be due to partial inhibition by Scytovirin, as it is known that slower growth increases capsule size (Garcia-Rodas et al., 2014). Finally, it is possible that for strain B3502, the binding of Scytovirin physically blocks vesicle release thereby explaining the observed decrease in capsule size. Elucidation of the binding partner(s) will aid in deciphering the process of capsule synthesis in *C. neoformans*, which currently constitutes a major knowledge gap in the field.

From this study, it is clear that the capsule is not the target for Scytovirin. Not only was binding localized to the cell wall, not the capsule, but the acapsular Cap67 strain was highly susceptible to Scytovirin inhibition. Moreover, synergy is noted with existing antifungals suggesting Scytovirin does not work through the same mechanisms. This suggests Scytovirin is recognizing a novel target, possibly an N-linked glycoprotein near or in the cell membrane or cell wall. This also indicates that Scytovirin will be effective against drug resistant pathogenic cryptococci. Since Scytovirin did not affect cell wall integrity on 0.5% Congo red, it suggests that the target of Scytovirin is not β-glucans, as expected from the carbohydrate specificity for high mannose moieties. Scytovirin also did not affect melanization of the various strains tested. Taken together, these data suggest that the target for Scytovirin is most probably a glycoprotein associated with the cell wall, not one involved in cell wall construction. Future experiments will be needed to identify the target and determine the mechanism of Scytovirin’s antifungal activity.

Understanding the differences among cryptococcal strains and serotypes at the molecular level is presently limited. Since the cell walls of the different serotypes are thought to have different compositions (Reese et al., 2007; McClelland and Casadevall, 2012), our results indicate that one potentially significant difference may be the presence and/or quantity of extracellular N-linked glycoproteins. Not only is there a significant difference in the potency for Scytovirin inhibiting serotype D versus serotype A strains, but there is also a significant difference between the serotype D sibling strains B3501 and B3502. Scytovirin susceptibility may be related to differences in virulence as B3501 shows high virulence and B3502 shows low virulence in mice (Nielsen et al., 2005). Thus, Scytovirin may serve as an important tool to characterize differences among cryptococcal strains on the level of glycan production and glycoprotein composition, as well as how glycan composition contributes to virulence. Understanding these differences is important and provides considerable insight into cryptococcal pathogenesis.

Overall, Scytovirin provides a promising new approach for cryptococcal inhibition. Further encouragement comes from multiple reports of favorable cytotoxicity for Scytovirin (Takebe et al., 2013; Garrison et al., 2014). Additionally, 70% of primary cutaneous cryptococcosis infections in Europe were found to be serotype D isolates (Neuville et al., 2003) adding potential for Scytovirin to be developed as an effective topical application. While Scytovirin itself appears promising, the novel findings presented herein can presumably extend to other high mannose binding lectins. Several high mannose binding lectins have positive cytotoxicity profiles and are under development as antivirals (O’Keefe et al., 2009; Huskens et al., 2010; Kouokam et al., 2011), including potential development as topical microbicides (Huskens and Schols, 2012), extending the future possibilities for this novel antifungal approach. Focusing on Scytovirin, structural engineering may lead to increased antifungal efficacy as reported for HIV-1 (McFeeters et al., 2013). The possibility also exists to utilize individual Scytovirin domains (Xiong et al., 2006), though it remains to be determined how carbohydrate specificity, affinity, and multivalency affect antifungal properties. Future studies are required to address such issues and will provide further understanding of the influence of lectins on the fungal life cycle. The novel antifungal function of Scytovirin reported herein indicates great potential for anticryptococcal development and that Scytovirin may serve as a tool to help understand differences in cryptococcal serotypes.

## Author Contributions

TJ and EM contributed equally to the work. TJ, EM, and HM contributed to data generation and collection. Specifically, TJ produced all Scytovirin. TJ and EM conducted all MFC measurements. TJ collected confocal localization data. EM collected all capsule release, capsule size, melanin, and cell wall stress data. HM and TJ conducted chitin binding experimentation. All authors contributed to experimental design, data interpretation, and data presentation. RM is primarily responsible for writing the manuscript, with significant contributions from EM and TJ and minor contribution and editing from HM.

## Conflict of Interest Statement

The authors declare that the research was conducted in the absence of any commercial or financial relationships that could be construed as a potential conflict of interest.
