# Supplementary material for: Novel Antifungal Activity for the Lectin Scytovirin: Inhibition of Cryptococcus neoformans and Cryptococcus gattii
Source: Front Microbiol. 2017 May 9;8:755. doi: 10.3389/fmicb.2017.00755 (PMC5422485; doi:10.3389/fmicb.2017.00755)
Supplement: Supplementary file 1 [file Data_Sheet_1.DOCX]

Supplementary Material

Novel Antifungal Activity for the Lectin Scytovirin: Inhibition of *Cryptococcus neoformans* and *Cryptococcus gattii*

Tyler H. Jones, Erin E. McClelland, Hana McFeeters, Robert L. McFeeters^*^

*** Correspondence:** Corresponding Author: [robert.mcfeeters@uah.edu](mailto:robert.mcfeeters@uah.edu)


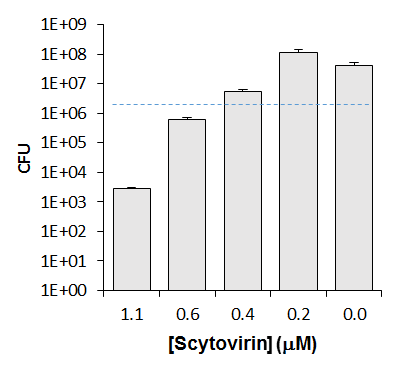

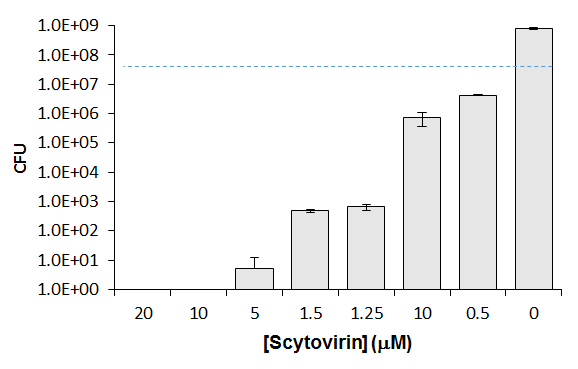


| [Scytovirin] | Average |  |
| --- | --- | --- |
| (μg/ml) | CFU/ml | StDev |
| 200 | 0 | 0 |
| 100 | 0 | 0 |
| 50 | 5.0E+00 | 7.1E+00 |
| 15 | 4.9E+02 | 5.7E+01 |
| 12.5 | 6.6E+02 | 1.6E+02 |
| 10 | 7.3E+05 | 3.8E+05 |
| 5 | 4.2E+06 | 1.3E+05 |
| 0 | 7.9E+08 | 5.4E+07 |

| [Scytovirin] | Average |  |
| --- | --- | --- |
| (μg/ml) | CFU/ml | StDev |
| 11 | 2.7E+03 | 3.0E+02 |
| 6 | 6.1E+05 | 1.3E+05 |
| 4 | 5.6E+06 | 5.4E+05 |
| 2 | 1.1E+08 | 3.8E+07 |
| 0 | 4.2E+07 | 8.8E+06 |

**Supplementary Figure 1.** CFU for Scytovirin MFC determination against *C. neoformans* strain 24067. The level used for MFC determination, 95% of the control with no Scytovirin present, is indicated by the horizontal dashed line. Tables with CFU counts and standard deviations are shown below the plots. StDev is the standard deviation and used for error bars.
