# Supplementary material for: Novel Antifungal Activity for the Lectin Scytovirin: Inhibition of Cryptococcus neoformans and Cryptococcus gattii
Source: Front Microbiol. 2017 May 9;8:755. doi: 10.3389/fmicb.2017.00755 (PMC5422485; doi:10.3389/fmicb.2017.00755)
Supplement: Supplementary file 2 [file Data_Sheet_2.DOCX]

Supplementary Material

Novel Antifungal Activity for the Lectin Scytovirin: Inhibition of *Cryptococcus neoformans* and *Cryptococcus gattii*

Tyler H. Jones, Erin E. McClelland, Hana McFeeters, Robert L. McFeeters^*^

*** Correspondence:** Corresponding Author: [robert.mcfeeters@uah.edu](mailto:robert.mcfeeters@uah.edu)

H99S

24067

B3502

**Supplementary Figure 2.** Oneway analysis of capsule diameter by [Scytovirin] is presented for *C. neoformans* strains H99S (top), 24067 (middle), and B3502 (bottom).
