# Supplementary material for: Novel Antifungal Activity for the Lectin Scytovirin: Inhibition of Cryptococcus neoformans and Cryptococcus gattii
Source: Front Microbiol. 2017 May 9;8:755. doi: 10.3389/fmicb.2017.00755 (PMC5422485; doi:10.3389/fmicb.2017.00755)
Supplement: Supplementary file 3 [file Data_Sheet_3.docx]

Supplementary Material

Novel Antifungal Activity for the Lectin Scytovirin: Inhibition of *Cryptococcus neoformans* and *Cryptococcus gattii*

Tyler H. Jones, Erin E. McClelland, Hana McFeeters, Robert L. McFeeters^*^

*** Correspondence:** Corresponding Author: [robert.mcfeeters@uah.edu](mailto:robert.mcfeeters@uah.edu)

| [AMB] | [Scytovirin] | Fractional Inhibition |
| --- | --- | --- |
| 0.031 | 0.391 | 0.519 |
| 0.016 | 0.195 | 0.462 |

The FIC index at 50% inhibition was calculated for these cells as follows:

| AMB |  | Scytovirin | FIC |
| --- | --- | --- | --- |
| 0.031 | + | 0.391 | 0.37 |
| 0.125 |  | 3.125 |  |
|  |  |  |  |
| AMB |  | Scytovirin | FIC |
| 0.016 | + | 0.195 | 0.19 |
| 0.125 |  | 3.125 |  |

The average of these FIC values, (0.37 + 0.19)/2 = 0.28, was rounded to 0.3.

**Supplementary Figure 3.** MFC-2 determination of FIC index. A checkerboard arrangement of varying drug concentrations was arranged in a 96 well plate. In the first column of the plate were varying concentrations of Scytovirin only (serial factor of 2 dilutions). In the first row of the plate, varying concentrations of AMB only (serial factor of 2 dilutions). The common cell had no Scytovirin or AMB. The concentration of Scytovirin alone causing the closest to 50% inhibition was 3.125 μg/ml, which demonstrated 57% inhibition. The concentration of AMP alone causing closest to 50% inhibition was 0.125 μg/mL which demonstrated 43% inhibition. Sorting the combined drug checkerboard data in terms of fractional inhibition (top entry), the FIC index at 50% inhibition was determined. All concentrations are expressed in μg/mL.
